# A New Sensorized Approach Based on a DeepLabCut Model and IR Thermography for Characterizing the Thermal Profile in Knees During Exercise

**DOI:** 10.3390/s24237862

**Published:** 2024-12-09

**Authors:** Davide Crisafulli, Marta Spataro, Cristiano De Marchis, Giacomo Risitano, Dario Milone

**Affiliations:** Department of Engineering, University of Messina, Contrada di Dio, 98166 Messina, Italy; dcrisafulli@unime.it (D.C.); cristiano.demarchis@unime.it (C.D.M.); giacomo.risitano@unime.it (G.R.); dmilone@unime.it (D.M.)

**Keywords:** infrared thermography, Wii Balance Board, DeepLabCut, diagnostic, sit-to-stand

## Abstract

The knee is one of the joints most vulnerable to disease and injury, particularly in athletes and older adults. Surface temperature monitoring provides insights into the health of the analysed area, supporting early diagnosis and monitoring of conditions such as osteoarthritis and tendon injuries. This study presents an innovative approach that combines infrared thermography techniques with a Resnet 152 (DeepLabCut based) to detect and monitor temperature variations across specific knee regions during repeated sit-to-stand exercises. Thermal profiles are then analysed in relation to weight distribution data collected using a Wii Balance Board during the exercise. DeepLabCut was used to automate the selection of the region of interest (ROI) for temperature assessments, improving data accuracy compared to traditional time-consuming semi-automatic methods. This integrative approach enables precise and marker-free measurements, offering clinically relevant data that can aid in the diagnosis of knee pathologies, evaluation of the rehabilitation progress, and assessment of treatment effectiveness. The results emphasize the potential of combining thermography with DeepLabCut-driven data analysis to develop accessible, non-invasive tools for joint health monitoring or preventive diagnostics of pathologies.

## 1. Introduction

The knee, as one of the most complex and vulnerable joints in the human body [1], is prone to various pathological conditions that can affect its surface temperature [2].

Such thermal variations can signify inflammatory processes, injuries, circulatory issues, or other abnormalities. Infrared Thermography (IR) is a non-invasive method used to map body thermal variations, providing valuable diagnostic and monitoring information. Several studies applied IR as a reliable technique to correlate variation in skin temperature to different knee pathologies [3,4,5]. Early diagnosis is crucial in degenerative knee processes, such as osteoarthritis, to increase the effectiveness of treatment. Carlin et al. [6] clinically evaluated the effectiveness of thermal infrared imaging in the diagnosis and evaluation of knee diseases. According to their results, thermography is a sensitive and reliable method for the diagnosis and monitoring of various knee pathologies, including osteoarthritis, rheumatoid arthritis, and ligament and tendon problems.

A recent study [7] analysed thermal normality patterns of the knee joint in professional athletes of different sports. The results showed thermal symmetry in the knee joint, both in the anterior and posterior region, with a contralateral skin temperature difference of less than 0.3 °C in elite athletes of judo, basketball, five-a-side football and volleyball with no symptoms of pain or injury. Anterior and posterior knee skin temperatures were similar between men and women. However, sports modalities showed significant differences in knee temperature and anterior-posterior thermal differences. The results highlight the importance of considering differences in motion modalities in the interpretation of thermographic results and in defining strategies to monitor and prevent injuries in athletes. Petrigna et al. [8] conducted a literature review on the use of thermography to assess a thermal attention threshold in people with knee osteoarthritis. Their study proposed a threshold value of 31.3 °C, suggesting that knee temperatures at or above this value could indicate the presence of osteoarthritis. However, the values are indicative and related to an analysis of a limited number of case studies. In fact, the conclusion states that there is a need for standardisation of testing protocols and data extrapolation due to the too many differences between studies on this topic, which makes it difficult to propose an unequivocal thermal threshold. In 2019, Payá et al. [9] explored the reliability of infrared thermography analysis of the patellar tendon. The focus has been dedicated to the location of landmarks and a region of interest (ROI) that can significantly affect the validity of the collected data.

The study of factors that can influence the accuracy of thermal acquisitions (i.e., [10]) has led over the years to the definition of some protocols for the use of thermography on the human body [11,12]. The definition of the area of interest (ROI) for temperature acquisition is not defined by standard protocols or guidelines, and the position, shape and size of the ROI in the thermogram may vary depending on the operator [13]. The use of external markers to define ROIs is one of the most reliable methods in the analysis of static conditions, whereas in case of analysis of dynamic situations, such as while walking or performing an exercise, the measurement is operator-dependent and often uses data averaged within a control area positioned around the zone of interest [14].

In recent years, the integration of artificial intelligence algorithms supports the development of new methods to improve diagnostic accuracy, treatment and research processes. In the diagnosis of knee pathologies, AI research has mainly focused on the development of neural networks to perform specific interpretative tasks, such as pathology detection, classification and segmentation [15,16,17,18,19,20]. Jin et al. [21] proposed an automated approach for the diagnosis of knee osteoarthritis using infrared thermography, dividing the knee area into several zones using the patella as a central reference. As a result, an automated system for thermal screening of the knee was achieved, improving clinical diagnosis.

The thermal behaviour of the knees is closely associated with weight distribution between the legs. Numerous studies have employed the Nintendo™ (Kyoto, Japan) Wii Balance Board™ (WBB) as an effective, low-cost alternative to traditional force plates in biomechanics and rehabilitation research. The WBB is particularly valuable for capturing vertical ground reaction forces and centre of pressure (CoP) data, which are used to evaluate weight bearing asymmetry even during dynamic exercises, such as gait and sit-to-stand cycles [22,23,24,25,26]. This type of data analysis is useful in identifying tendencies such as increased reliance on one leg or shifts in balance, which are critical for clinical assessments of functional ability and fall risk, particularly in aging populations [23,26]. The WBB’s efficiency and reliability have been thoroughly validated in the literature, with studies comparing its measurements to those obtained from standard force plates in medical practice [22,24,25,27,28].

In their 2010 study, Clark et al. [28] evaluated the validity and reliability of the WBB as a tool for assessing standing balance. The research involved thirty participants without lower limb pathologies, who performed single-leg and double-leg standing balance tests under eyes-open and eyes-closed conditions on two separate occasions. To determine the WBB’s accuracy, its measurements of center of pressure (COP) path length were compared to those obtained using a laboratory-grade force platform (FP), considered the gold standard. Both devices demonstrated strong reliability, with within-device test-retest intraclass correlation coefficients (ICCs) ranging from 0.66 to 0.94 and between-device ICCs ranging from 0.77 to 0.89 across all testing protocols.

These comparisons consistently demonstrate that the WBB provides a robust and accurate measure of postural control and balance parameters, supporting its integration into both research and clinical applications.

This study proposes a new protocol for detecting temperature profiles in various areas of the knee during exercise, aimed at diagnosing potential knee injuries, monitoring disease progression, and evaluating treatment effectiveness. This will be achieved through the combined use of a model trained with DeepLabCut and the infrared thermography technique. The thermal trends were correlated to the weight distribution, evaluated through the WBB acquisition during the sit-to-stand cycles. The differences between the temperature profiles were highlighted by comparing the use of semi-automatic and automatic data extrapolation using a Resnet 152 (DeepLabCut-based). This approach combines different methodologies and could find several potential biomedical applications, as it has numerous advantages. These include non-invasive operation, not requiring the application of external markers to ensure position measurement accuracy, and the ability to be used with a large dataset.

## 2. Materials and Methods

A new methodology for detection of the temperature profile on different knee areas during a sit-to-stand exercise is described in this section. Throughout the test, data on weight distribution across the feet were recorded to analyse their correlation with temperature patterns on the knee surface.

Data acquisition of thermal images was performed at the frequency of 20 Hz using the Radiamatic Timage XT infrared camera (IRtech, E instrument Group, Lesmo, Monza Brianza, Italy) equipped with a microbolometer sensor that had an optical resolution of 382 × 288 pixels and a thermal sensitivity of 0.04 °C. Thermal images were processed with the proprietary Timage Connect camera software, Matlab^®^ (The MathWorks Inc., R2024b, Natick, MA, USA) and a deep learning algorithm.

The evaluation of the weight distribution during the exercise was performed using a Nintendo™ (Kyoto, Japan) Wii Balance Board™ (WBB) with four load cells, two for each foot. Data on the participant’s weight distribution were collected at 20 Hz and transmitted to a computer via Bluetooth. As stated in the introduction, the scientific literature highlights numerous applications utilizing the WBB as an accessible, portable, low-cost tool for assessing balance in patients, demonstrating its reliability in contexts where a high degree of accuracy is not required [29,30]. Rohof et al. [31] assessed the performance of the (WBB) in a study involving 41 healthy subjects, utilizing the one-leg stance yoga pose “Tree” and the balance game “Table Tilt”. Results were compared with two established systems, the MFT-S3 Check and the Posturomed. Their findings indicate that data acquisition with the WBB is comparable to that of laboratory-grade force platforms, confirming its suitability for evaluating postural stability.

No filtering was applied to the data collected from the Wii Balance Board. Conversely, the thermal data were processed using a rowess filter with a 2% span to suppress outlier spikes and highlight the overall temperature trend. Synchronization between the weight distribution data and knee temperature variations was achieved by aligning them to the test execution timeline.

### 2.1. Cohort

Twenty healthy volunteers participated in this study with no history of knee pain, lower limb surgery or lower limb injuries in the last six months. The sample consists of 14 males and 6 females aged between 22 to 44 years (age = 27.4 ± 5.1 years, body weight = 69.1 ± 14.5 kg, height = 163.0 ± 38 cm). Among the participants, 11 showed a BMI in the 18.5–24.9 kg/mm^2^ range while the remaining 9 showed a BMI between 25 and 30 kg/mm^2^. All subjects provided written informed consent, all the experiments were carried out according to the principles of the Declaration of Helsinki, and the participants declared that they had not consumed caffeine or alcohol in the 48 h prior to the experiment to avoid inducing changes in heart rate and blood pressure [32].

### 2.2. Procedure

Several factors can influence the results of the thermal acquisition during the test. For this reason, to maximize the reliability of this research activity, the previous mentioned guidelines [11,12] have been followed, particularly referring to the room environmental conditions and equipment requirements.

**Figure 1:**
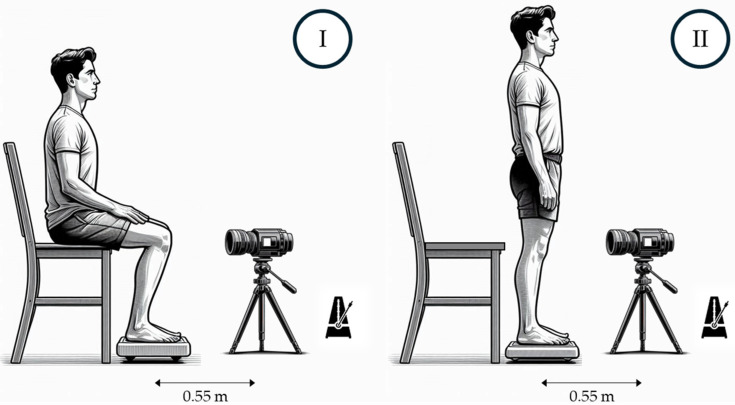
Schematic representation of the procedural setup, equipment (WBB, infrared camera and metronome) and sit-to-stand phases: (**I**) sitting phase; (**II**) standing phase.

A schematic of the setup configuration is provided in the Figure 1, detailing the equipment used and the phases of the sit-to-stand exercise.

The study was carried out in an air-conditioned room with controlled temperature and humidity. During the tests, the average temperature was 27 °C with 60% relative humidity. For the test, an infrared camera, fixed on a tripod, was used to acquire thermographic images of the region between the patients’ patellar tendon and quadriceps tendon. The camera was positioned at a fixed distance of 0.55 m from the patient, measured at ground level by taking the centre of the WBB and the centre of the tripod as reference. The height of the camera was adjusted according to the anatomical characteristics of each participant to ensure optimal focus on the area of interest. Prior to starting measurements, the patient stood on the WBB, which was calibrated using known weights to ensure accuracy. The participants were asked to wear shorts to ensure the area being analyzed remained exposed. Upon entering the room, participants remained seated at rest for 20 min to allow for thermal stabilization, enabling their body temperature to reach equilibrium with the room environment.

**Figure 2:**
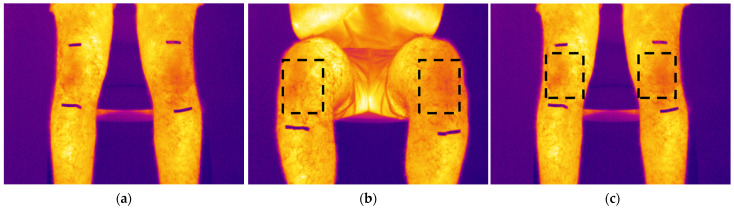
Thermograms during a sit to stand exercise phase: (**a**) Standing position without ROI; (**b**) Sitting position with ROI; (**c**) Standing with ROI.

After this pre-activity phase, participants began the exercise from a sitting position (Figure 1—Phase I), performing repeated cycles of standing up (Figure 1—Phase II) and sitting down for ten minutes. To maintain a consistent exercise pace, a metronome was used, guiding each sit-to-stand cycle (Phase I—Phase II—Phase I) to be completed every 3–4 s. To ensure precise identification of the region of interest (ROI) during the analysis phase, metal markers were applied to the subjects’ legs, approximately one in the lower end of the quadriceps tendon and the other in the lower end of the patellar tendon (Figure 2a). Thermographic images were acquired, ensuring that the area delimited by the markers was fully visible and centred. These markers aid in identifying the region of interest (ROI), enabling comparison across different thermograms during manual data extraction. Previous studies have shown that the use of such markers can improve the accuracy of ROI detection, contributing to greater reproducibility and reliability of results [9].

### 2.3. Semi Automatic Post Processing Methodology

In the semi-automatic post-processing phase, an initial sampling of thermal data acquired via infrared camera has been performed, considering one thermogram every 30 s during a 10-min activity, resulting in a total of 20 frames for each participant. Following this, a rectangular area within the region defined by the metal markers is employed to extract both maximum and minimum temperature values (Figure 2b,c). Frame by frame, the rectangular box had to be adjusted and redefined according to the current position of the knee regions during the movement from sitting to standing.

### 2.4. Automatic Post Processing Methodology

**Figure 3:**
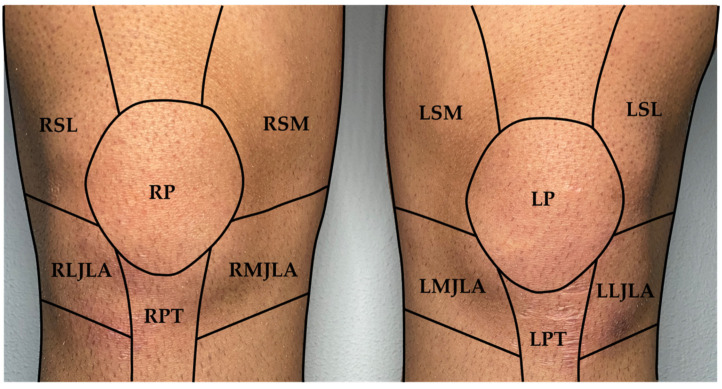
Named knee zones and boundaries: SL, superior lateral; SM superior medial; P, patella; LJLA lateral joint line area; MJLA, medial joint line area; PT, patella tendon. Initial letters: R, right leg; L, left leg.

The automatic post processing pipeline is described in detail in the next chapter. The different zones of interest of the knee have been defined as reported in Figure 3, inspired by the photographic knee pain map proposed by Elson et al. [33]. On each knee, six zones have been defined; superior lateral (LS), superior medial (SM), patella (P), lateral and medial joint line areas (LJLA and MJLA) and patella tendon (PT). Each acronym is preceded by the letter ‘R’ or ‘L’ to designate the right or left leg, respectively.

### 2.5. Overview of DeepLabCut

DeepLabCut (v2.3.10) is a software toolbox designed for markerless motion capture using deep learning algorithms [34,35,36]. It provides a framework that allows researchers to track and analyze the movement of animals or humans from video data without the necessity of attaching physical markers to subjects. This is achieved through a process where neural networks are trained to recognize and follow key points on the body, even in complex and varied environments. DeepLabCut’s approach is based on transfer learning, meaning it can be trained with relatively few annotated frames and still achieve high accuracy in pose estimation across diverse subjects and settings. Motion capture systems like DeepLabCut are especially useful for studies where traditional marker-based techniques are impractical or intrusive. By using image-based tracking, it captures detailed motion data that is valuable in fields such as neuroscience, ethology, and biomechanics. The flexibility of this system makes it suitable for both laboratory and naturalistic settings, accommodating a wide range of experimental conditions and movement types [37,38,39,40].

The underlying architecture of DeepLabCut often employs deep residual networks (such as ResNet), which are crucial for handling the challenges associated with training deep neural networks. Specifically, the software employs a ResNet-152, which is a deep residual network known for its exceptional performance in neural networks. As outlined in the ResNet architecture, this deeper variant of ResNet utilizes a bottleneck design that incorporates three layers for each residual function 1 × 1, 3 × 3, and another 1 × 1 convolution where the 1 × 1 layers play a vital role in dimension reduction and restoration [41,42]. This advanced configuration addresses issues such as the vanishing gradient problem, making it possible to train deeper networks and improve pose estimation accuracy. By requiring input image dimensions that are multiples of 32 and using a 7 × 7 convolutional layer followed by max pooling, DeepLabCut leverages these structural advancements to efficiently process images at high resolution.

This design allows the software to maintain performance while analyzing high-resolution images, ensuring that critical details in the motion data are not lost. The ability to capture and analyze motion with such precision allows DeepLabCut to be used in other domains, such as thermographic imaging. Here, its capacity to track body regions could be leveraged to estimate thermal data points accurately. By applying this method, researchers could segment and analyze temperature variations over time in a non-invasive way, offering a new approach to studying physiological processes linked to movement and heat distribution.

#### DeepLabCut Model Performance

The videos were manually labeled according to the regions described in Figure 3. For each video, significant frames were extracted using the k-means algorithm to avoid the issue of labeling the same data twice. Subsequently, 80% of the dataset was used by DeepLabCut for training purposes with the remaining 20% for validation. The training process, outlined in Table 1, involved multiple iterations, each defined by distinct dataset sizes, shuffle parameters, and precision metrics. Data were reported iteratively until the identified parameters no longer showed variation, ensuring that the model had reached a stable state.

Table 1 provides an overview of the key metrics from the training process, demonstrating the stabilization of model performance:

- Train and Test Errors: Both training and testing errors, measured in pixels, showed a steady decrease across iterations before reaching stability. The training error reduced from 11.6 px in the first iteration to 9.6 px in the final iteration, while the test error decreased from 11.8 px to 9.3 px.
- p-cut Adjusted Errors: Errors recalculated using the p-cut threshold (p-cut = 0.5) followed a similar trend, with the train error under p-cut decreasing from 7.3 px to 5.8 px and the test error dropping from 7.4 px to 5.7 px.

These results highlight the refinement of the model’s performance, with iterations continuing until the parameters stabilized and no longer exhibited changes. This demonstrates the robustness of the training process, and the reliability of the methodology applied.

### 2.6. Overview of Statistical Analysis 

To present the findings and compare the semi-automatic and automatic methods for temperature extraction, a statistical analysis using MATLAB was conducted. Bar charts were generated to evaluate the number of participants showing a correlation between the leg bearing the greater load and the leg with the most significant temperature variation, based on the results from both methodologies. Additionally, the extracted thermal data, averaged over the region of interest (ROI), was compared to data from a specific area of the knee. Specifically, a linear model was employed as correlation analysis between the maximum temperature extracted using the semi-automatic method and the temperature recorded at the medial collateral ligament using the proposed automatic method. A similar analysis was conducted for the minimum temperature, comparing it with the temperature measured at the patella. Classifications were performed based on gender (male-female), body mass index (BMI; normal weight < 18.4, overweight > 18.4), and the presence of weight asymmetry (one leg bearing > 52% of the weight). Finally, the Bland–Altman analysis was conducted to assess the agreement between two measurement methods across four regions: Right Patella, Left Patella, Right Ligament, and Left Ligament.

## 3. Results

Combined thermographic analysis and Deep learning techniques (Deeplabcut trained model) enabled the assessment of thermal profiles across various knee regions during repeated sit-to-stand exercises. Specifically, evolving knee temperature profiles were captured during 10-min cycles of sit-to-stand exercises. Two post-processing methodologies—semi automatic and automated—were investigated and are detailed in the following sections. The thermal profile trends on participants’ knees were additionally correlated with weight distribution assessments between the legs, measured using the WBB.

### 3.1. Temperature Assessment

**Figure 4:**
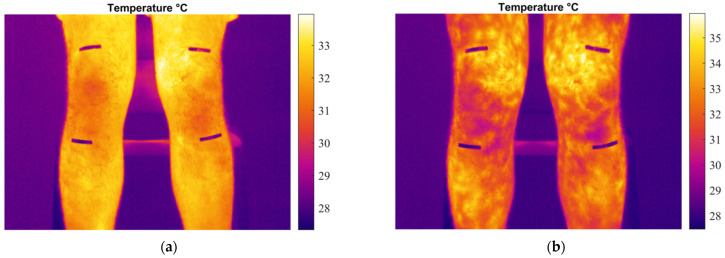
Temperature map of the knee region: (**a**) pre-exercise; (**b**) post-exercise.

The physical activity produces a generalized temperature modification on the body, related to a variation on skin blood flow [43]. Figure 4 shows a visible variation in the temperature distribution in the legs between the two thermograms of a participant before and after performing the exercise.

### 3.2. Semi Automatic Post-Processing

The semi-automatic post-processing method for thermal profile extraction consisted of positioning a rectangular ROI frame by frame on the knee region, as shown previously in Figure 2. For all participants, maximum and minimum temperature from each frame have been extracted from the ROI, since the highest temperature consistently appeared in the region of the medial collateral ligament, while the lowest temperature was observed in patella or patellar tendon area. These findings align with the impact of physical exercise on skin microvascular responsiveness, which appears to be greater in the medial collateral ligament region. This heightened responsiveness is reflected in the consistently higher temperatures observed in this area, compared to the lower temperatures recorded in the patellar tendon region [44].

**Figure 5:**
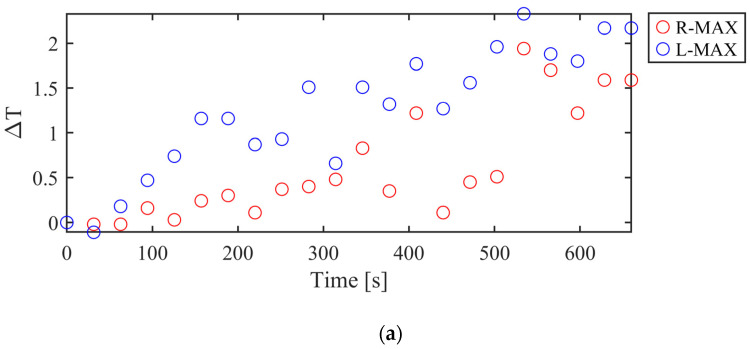

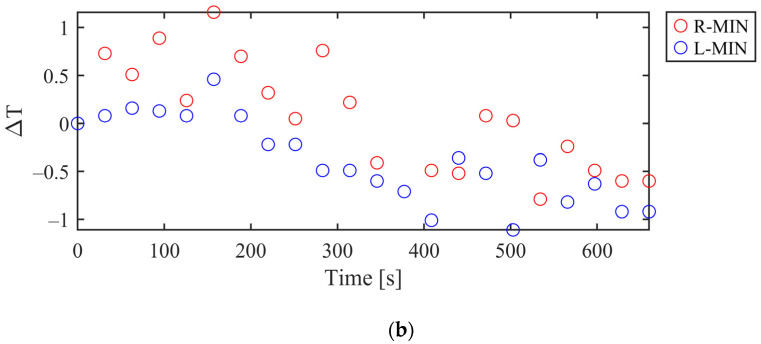
Temperature variation during exercise for the right (red) and left (blue) legs is shown for: (**a**) maximum temperature trend and (**b**) minimum temperature trend.

The thermal profile of one participant has been reported in terms of temperature variation over the time (Figure 5). In the graph, red markers indicate the right leg, whereas blue markers represent the left leg.

During the test, maximum temperature consistently displayed an increasing trend, while minimum temperature generally exhibited a decreasing trend in most cases, according to the increasing blood flow to the active muscle as stated by Simmons et al. [43].

### 3.3. DeepLabCut Post-Processing

The automation of temperature data extraction for specific knee regions was developed using the thermal dataset acquired as described in Section 2. Automating the process through the DeepLabCut model allows for efficient processing of large datasets to determine a position of a specific target over the time.

**Figure 6:**
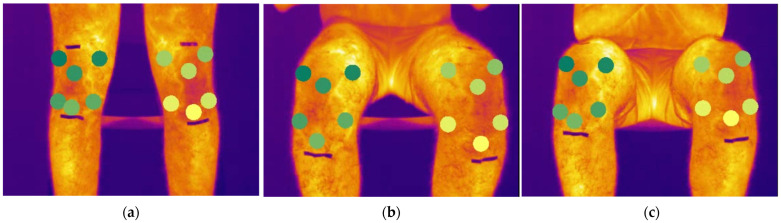
Thermograms during a sitting phase, green circles represent the different regions detected by the DeepLabCut model on the knee area on the standing phase (**a**), during sitting phase (**b**) and sitting (**c**).

In this study, one image per second was provided as input to the Resnet 152 model for each participant over 10 min of exercise (approximately 600 images per participant), resulting in output pixel coordinates marking the centre of each knee region defined in Figure 3 for every frame. The neural network identified the various knee regions throughout all phases of movement, as shown in Figure 6, with an average likelihood function of 0.99 for all case studies. Temperature values for each knee area were subsequently extracted from these pixel coordinates across the entire exercise duration.

**Figure 7:**
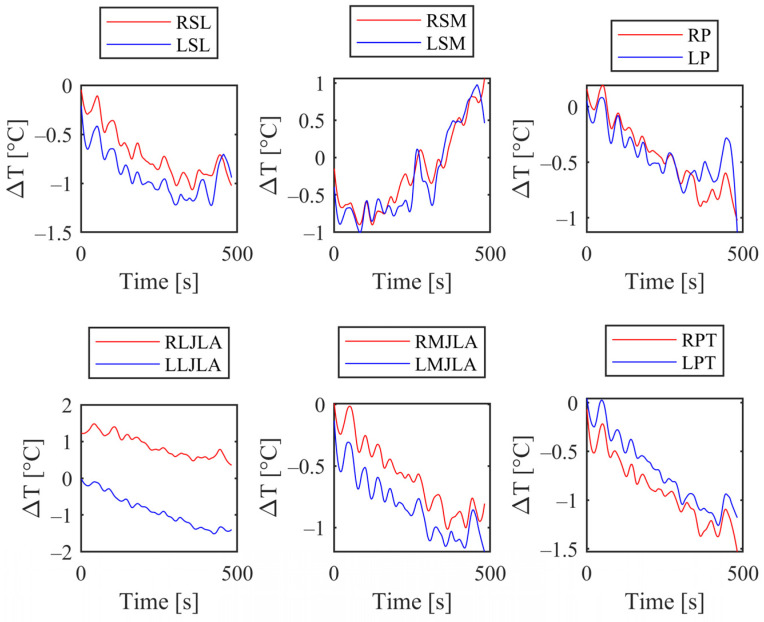
Temperature evolution on knee areas showed in Figure 3.

The temperature profiles extracted with the automatic post-processing methodology for each knee zones have been reported in Figure 7.

As previously observed during the semi-automatic post-processing phase, a notable temperature increase is associated with the region encompassing the medial collateral ligament. This observation is further supported by the temperature trend analysis across different knee areas, which also highlights a significant increase in the medial collateral ligament region (see Figure 7, RSM and LSM curves).

The results for the patellar region and patellar tendon (RP and LP, RPT and LPT) showed a decrease in temperature as indicated in the previous paragraph.

No evidence of correlation patterns emerged from the comparison of the other different temperature trends of the knee areas of the participants.

### 3.4. Weight Distribution Evaluation

This section presents the results of data acquisition from the WBB. Assessing asymmetries in body weight distribution between the legs could impact the thermal profiles extracted from knee regions, depending on the leg analysed. Therefore, this study evaluated the distribution of weight between the legs, as well as between the forefoot and rearfoot, and continuously monitored the centre of pressure (CoP) position throughout the exercise. By tracking these variables during the sit-to-stand cycles, it is possible to account for potential influences of weight distribution on knee temperature profiles, thereby enhancing the precision of thermal assessment and its relevance to knee joint health.

**Figure 8:**
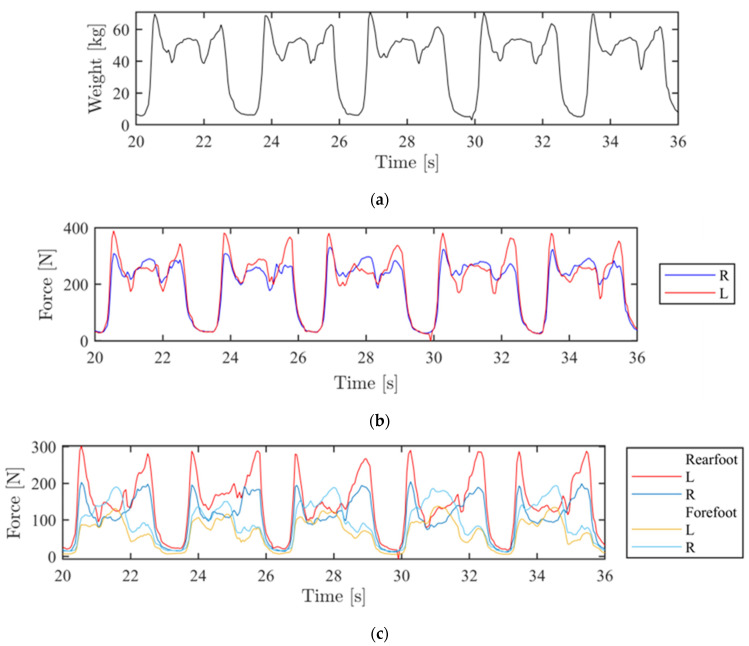
Wii Balance Board acquisition from 20 to 36 s of the sit-to stand activity: (**a**) Weight distribution (kg); (**b**) Force distribution on right (blue) and left (red) leg; (**c**) Force distribution on left (red) and right (blue) rearfoot, left (yellow) and right (cyan) forefoot.

Figure 8 depicts the progression of data collected from the four load cells throughout the activity. For clarity and readability, the results are displayed over a reduced time interval (from 20 to 36 s), focusing on a subset of sit-to-stand cycles.

Figure 8a illustrates the trend in total weight distribution over the selected time interval. When the participant stands, the weight is fully distributed into the feet, resulting in an increase in weight recorded by the WBB. During this phase, the value stabilizes around a level corresponding to the participant’s static weight. In the sitting phase, weight is transferred to the chair, leading to a decrease in the graph curve until reaching a minimum value near zero, indicating that nearly all weight is transferred to the chair.

Figure 8b illustrates the distribution of weight force over the selected time interval, divided between the legs. Here, a slight tendency of the participant to exert more force on the left leg while rising is observed. In general, patients did not exhibit an asymmetry in weight distribution between the legs exceeding 54.5% during exercise performance. Specifically, 13 out of 20 participants (65%) demonstrated a weight distribution of at least 52% on one leg during the exercise. Interestingly, this patient group exhibited more pronounced warming of the medial collateral ligament in the leg bearing greater weight compared to the leg with less load.

Figure 8c shows the distribution of force between the forefoot and rearfoot for both legs over the same time interval. In all participants, it was noted that the rearfoot carries the greatest load when in the standing position.

**Figure 9:**
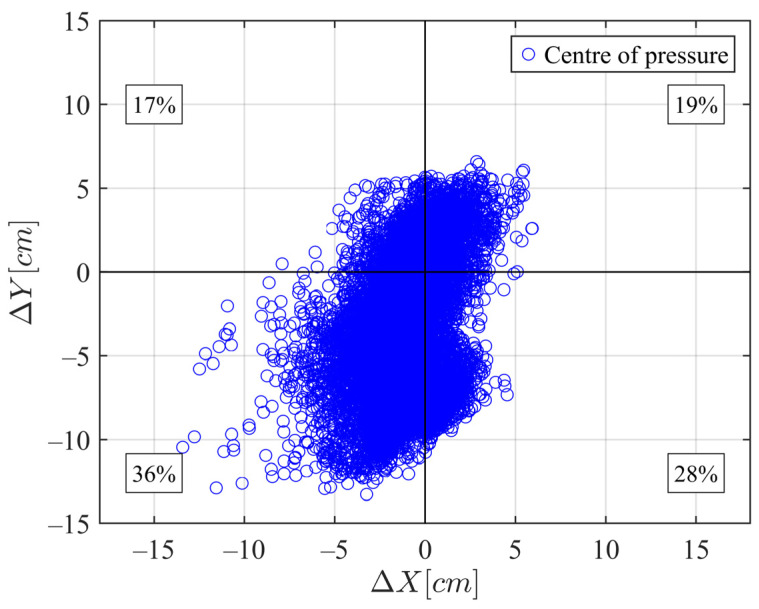
Centre of pressure position and body weight distribution on the feet for each of the four loadcells (in %) during the exercise.

Data on the position of the centre of pressure (CoP) were assessed using the WBB during the exercise (Figure 9). The coordinate point (0, 0) represents the centre of the WBB, with the *x*-axis indicating medio-lateral direction displacement and the *y*-axis representing antero-posterior direction movement of the subject. The percentages in each corner quantify body weight distribution across the four load cells. Over time, the trajectory of the CoP position forms an elongated shape aligned with the direction of movement. Additionally, the weight force is predominantly exerted on the rearfoot, with a slight imbalance toward the left side.

### 3.5. Statistical Analysis

**Figure 10:**
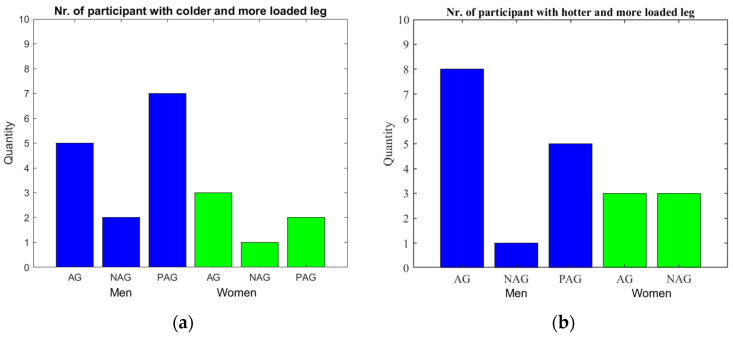
Correlation between the leg bearing the greater load and the leg with the most significant thermal variation classified by gender (men = blue, women = green) (AGREE (AG) true for both methods, DISAGREE (DAG) false for both methods, PARTIAL AGREEMENT (PAG) true for one of the two methods): (**a**) Leg with the greatest cooling; (**b**) Leg with the greatest heating.

In this section, the results of statistical analysis are presented. Specifically, the number of participants showing agreement between the leg bearing the greater load and the leg exhibiting the highest increase or decrease in temperature was examined. This evaluation was carried out by considering both the results of the semi-automatic method and those of the automated method, and the results have been displayed in the bar charts of Figure 10. In the graphical representation, male participants are indicated in blue and female participants in green. For each gender, the measurements classified as “AGREEMENT” (AG) indicate that both methodologies produced consistent results, demonstrating a correlation between the leg bearing the greater load and the leg exhibiting either increased heating of the medial collateral ligament or increased cooling of the patella.

Measurements classified as “DISAGREEMENT” (NAG) reflect cases where the two methods produced the same non-matching results. For example, for males, one case showed no correlation between the most loaded leg and the leg with the greatest thermal variation, while for females, this discrepancy occurred in three cases (Figure 10b).

Conversely, the measurements categorized as “PARTIAL AGREEMENT” (PAG) represent instances where one method identified a correlation between the most loaded leg and the leg with the most significant thermal variation, while the other method did not.

**Figure 11:**
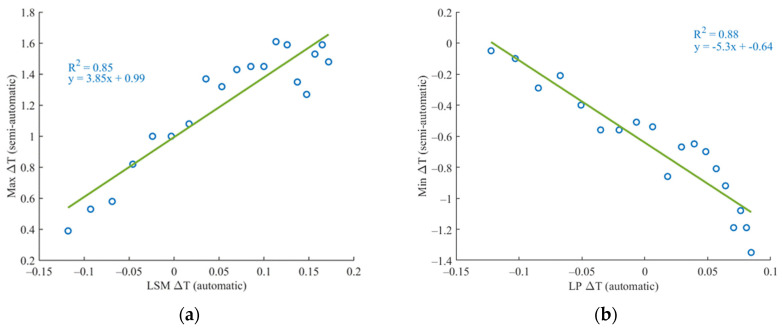
Regression analysis between the semi-automatic method and the automatic one. A strong correlation was found for 67% of the measurements with R^2^ of 0.71 ± 0.17, both for the ligament (**a**) and the patellar zone (**b**).

The linear regression model was applied to evaluate the interclass correlation between temperatures obtained using semi-automatic and automatic methods. The results have been further distinguished by gender, BMI and asymmetries in body weight distributions. Since the automatic methods provide continuous, real-time data, while the semi-automatic method captures only 20 discrete measurements, the automatic data was interpolated to match the specific timestamps of the semi-automatic readings. Following this adjustment, the correlation between the methods was analysed along with the coefficient of determination (R^2^). Across 80 measurements, 67% demonstrated a strong correlation with an average R^2^ of 0.71 ± 0.17, indicating high agreement between the methods (Figure 11).

**Figure 12:**
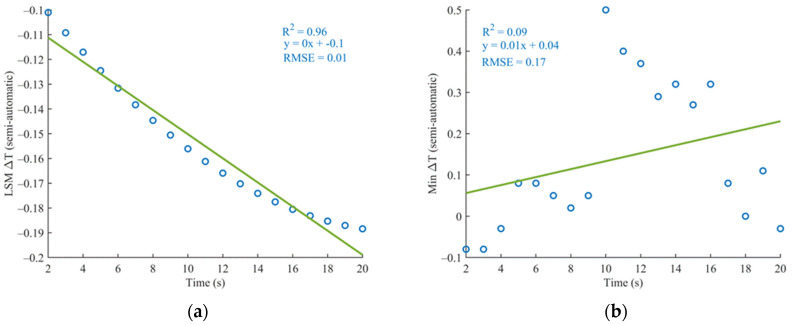
Thermal trend in the automatic (**a**) and semi-automatic (**b**) methods shows distinct differences. The automatic shows a clear decreasing trend, with an RMSE of 0.01, indicating a near-perfect linear trend. In contrast, the semi-automatic method lacks a significant correlation, reflected by a much higher RMSE of 0.15.

As shown in Table 2, the results indicate that women, participants with a normal weight, and those with unbalanced conditions exhibited slightly higher correlations compared to other groups. The remaining 33% of the total measurement exhibited a weak correlation, with an average R^2^ of 0.12 ± 0.08, suggesting little to no agreement. Among the strongly correlated measurements (67%), a further analysis was performed based on gender and other participant characteristics. Investigating the lack of correlation in these cases, the issue appears to stem from the quality of the thermal data obtained through the semi-automatic acquisition method. This hypothesis is supported by the higher RMSE (root mean square error) values observed in the temperature-time trends of the semi-automatic method compared to those of the automatic method, indicating greater variability and reduced reliability in the semi-automatic measurements (Figure 12).

**Figure 13:**
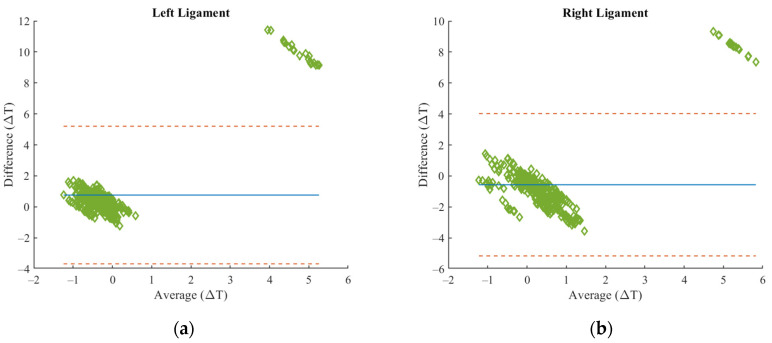

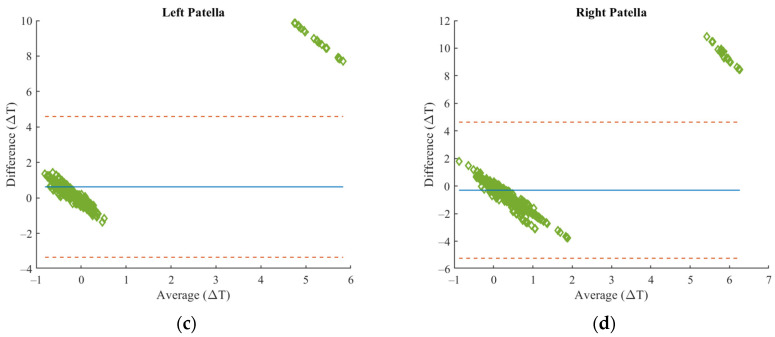
Bland–Altman plots of Left and Right Ligament (**a**,**b**), Right Patella, and Left Patella (**c**,**d**). The green diamonds represent the individual data points, plotting the difference between the two methods on the y-axis against the average of the two measurements on the x-axis. The blue line indicates the mean difference between the two methods. The red dashed lines represent the 95% confidence range, within which most of the data points are expected to fall.

The Bland–Altman test was conducted to assess the agreement between the two methodologies across the four regions of Right Patella, Left Patella, Right Ligament, and Left Ligament. The results show notable differences in agreement depending on the anatomical region (Figure 13).

Overall, the ligament regions (Right and Left) exhibited better agreement between the methods, with minimal bias and tighter limits of agreement. In contrast, the patella regions (Right and Left) showed larger variability, wider limits of agreement, and occasional outliers, suggesting less consistent agreement, particularly at higher measurement values.

## 4. Conclusions and Discussion

This study introduces a novel methodology for monitoring temperature variations across distinct knee regions during repetitive exercise cycles, alongside tracking weight distribution over time. This dual approach enables the identification of postural imbalances and explores potential correlations between asymmetric weight-bearing and abnormal temperature variations in the legs.

The cyclic execution of the sit-to-stand exercise was observed to result in localized warming of the medial collateral ligament, while the patella and patellar tendon tended to exhibit a cooling effect in most cases. This distinct thermal response pattern may reflect the different mechanical demands placed on different knees during repetitive weight-bearing transitions, highlighting the influence of the exercise on joint thermoregulation. Specific warming of the medial collateral ligament is related to the nature of the exercise; in fact, the squat is often used to assess recovery of the medial collateral ligament following surgical intervention [45].

Additionally, comparison of knee temperature trends with weight distribution data revealed that greater warming of the medial collateral ligament was associated with the leg bearing a higher load. Specifically, in participants who demonstrated an asymmetrical weight distribution, with more than 52% of their body weight on one leg (13 out of 20 participants), the increased thermal response of the medial collateral ligament corresponded to the more heavily loaded leg. This finding suggests a relationship between mechanical load and localized heat generation in knee areas, providing insights into how load-bearing influences knee temperature dynamics during repetitive motion. No clear association was identified between temperature trends in other knee regions and weight distribution patterns. The primary aim of this study was to introduce a novel method for analysing thermal responses during movement; however, further research is needed to better understand the thermal behaviour across various knee areas. When comparing the two methodologies for assessing the match between the leg bearing the greater load and the leg exhibiting the highest increase or decrease in temperature, gender differences were evaluated. Among male participants, the results were more definitive in detecting trends in maximum temperature variations, whereas greater uncertainty was observed in the evaluation of minimum temperature changes. This discrepancy may be attributed to the fact that thermal variations associated with heating are more pronounced and easier to detect compared to those related to cooling, which tend to be less distinct. For female participants, however, no significant conclusions could be drawn due to the smaller sample size relative to male participants, limiting the statistical power of the analysis. A linear regression model was employed to evaluate the interclass correlation between temperatures obtained using the semi-automatic and automatic methods. The automatic method, which provides continuous real-time data, was compared to the semi-automatic method, which captures 20 discrete measurements. To ensure compatibility, the automatic data was interpolated to align with the specific timestamps of the semi-automatic readings. The correlation between the two methods was then analysed, and the strength of the relationship was quantified using the coefficient of determination (R^2^), providing insight into the consistency and reliability of the methods.

The results demonstrate the capabilities of both semi-automatically processed and DeepLabCut-driven automated methods, highlighting the advantages and limitations of each. Semi-automatic data processing involves repetitive steps, requiring operators to select and analyse individual frames to export results. While increasing the sampling rate of thermal data (up to the infrared camera’s acquisition frequency) can improve accuracy, this approach is time-consuming, as operators must manually define regions of interest (ROIs) for each frame. In contrast, the automated system proved to be a reliable tool for identifying knee regions to extract temperature data accurately. Due to its DeepLabCut-driven nature, the system can handle large input datasets without excessive processing time or the risk of operator errors inherent in repetitive tasks. This scalability and reliability underscore its utility for extensive thermal monitoring applications without compromising accuracy or efficiency. Despite the majority of the measurements showing a strong correlation between the two methods (R^2^ = 0.71 ± 0.17), a weak correlation was observed in 33% of them. This is regarding the quality of thermal data obtained via the semi-automatic acquisition method. This hypothesis is supported by higher RMSE values observed in the temperature-time trends of the semi-automatic method, indicating greater variability and reduced reliability compared to the automatic method.

The Bland–Altman analysis revealed variations in agreement between the two methods depending on the anatomical region. The patella regions showed less consistent agreement, with wider limits of agreement, greater variability, and systematic biases, particularly at higher temperatures. In contrast, the ligament regions exhibited stronger agreement, with near-zero biases, narrower limits of agreement, and minimal variability. These results highlight better reliability of the methods in ligament regions compared to patella regions.

The thermal response in the human body is closely tied to exercise type. For instance, repetitive movements like stepping up and down, treadmill walking, or cycling activate different muscle groups and lead to varying heat emission patterns. Therefore, future research could explore the thermal responses of various body regions under different types of exercises to gain more insights into muscle activation and heat distribution. Moreover, future studies could investigate the influence of dietary habits, hormonal levels and metabolic rate to identify potential correlations with temperature variations and their underlying physiological mechanisms.

Although all participants in the present study reported no known knee issues, future research could focus on individuals with diagnosed knee pathologies. This would enable a comparison of thermal and weight distribution responses between those with knee conditions and healthy participants, potentially uncovering differences in temperature variations and load distribution patterns associated with specific knee disorders. Such findings could significantly enhance rapid diagnostic techniques, injury prevention, and the monitoring of recovery progress post-surgery. Furthermore, these applications could be extended beyond the knee to other commonly injured areas, such as the ankle, elbow, and shoulder, particularly in contexts involving physical activity.

## Figures and Tables

**Table 1:** Training results of DeepLabCut model.

Iter.	Train Iter.	Dataset (%)	Shuffle	Train Error (px)	Test Error (px)	p-Cut	Train Err (p-Cut)	Test Err (p-Cut)
0	25 k	80	1	11.6	11.8	0.5	7.3	7.4
1	250 k	80	1	10.6	10.5	0.5	6.3	6.5
2	300 k	80	1	9.6	9.3	0.5	5.8	5.7

**Table 2:** Correlation analysis between the semiautomated and automated methods.

		Correlated Cases (67.5%)	W	M	BMI > 18.4	BMI < 18.4	Balanced	Not Balanced
R^2^	Mean	0.71	0.72	0.68	0.66	0.72	0.69	0.71
	Std	0.17	0.17	0.2	0.21	0.16	0.21	0.16

## Data Availability

The data presented in this study are available on request from the corresponding author.
